# Grating-coupled interferometry reveals binding kinetics and affinities of Ni ions to genetically engineered protein layers

**DOI:** 10.1038/s41598-020-79226-w

**Published:** 2020-12-17

**Authors:** Hajnalka Jankovics, Boglarka Kovacs, Andras Saftics, Tamas Gerecsei, Éva Tóth, Inna Szekacs, Ferenc Vonderviszt, Robert Horvath

**Affiliations:** 1 Bio-Nanosystems Laboratory, Research Institute of Biomolecular and Chemical Engineering, University of Pannonia, Egyetem u. 10, Veszprém, Hungary; 2 Nanobiosensorics Laboratory, Institute of Technical Physics and Materials Science, Centre for Energy Research, Konkoly-Thege Miklós út 29-33, Budapest, Hungary

**Keywords:** Biophysics, Nanoscale biophysics, Optics and photonics, Applied optics, Optical sensors, Metals, Biomarkers

## Abstract

Reliable measurement of the binding kinetics of low molecular weight analytes to their targets is still a challenging task. Often, the introduction of labels is simply impossible in such measurements, and the application of label-free methods is the only reliable choice. By measuring the binding kinetics of Ni(II) ions to genetically modified flagellin layers, we demonstrate that: (1) Grating-Coupled Interferometry (GCI) is well suited to resolve the binding of ions, even at very low protein immobilization levels; (2) it supplies high quality kinetic data from which the number and strength of available binding sites can be determined, and (3) the rate constants of the binding events can also be obtained with high accuracy. Experiments were performed using a flagellin variant incorporating the C-terminal domain of the nickel-responsive transcription factor NikR. GCI results were compared to affinity data from titration calorimetry. We found that besides the low-affinity binding sites characterized by a micromolar dissociation constant (*K*_d_), tetrameric FliC-NikR_C_ molecules possess high-affinity binding sites with *K*_d_ values in the nanomolar range. GCI enabled us to obtain real-time kinetic data for the specific binding of an analyte with molar mass as low as 59 Da, even at signals lower than 1 pg/mm^2^.

## Introduction

In the last few decades, label-free optical biosensors have proven their unique capabilities in a wide range of applications from biomolecular interaction analysis (BIA)^1–4^ to whole-cell monitoring^5–9^. Screening the interactions of various analyte-ligand pairs has become essential in drug discovery^10–13^, assay and sensor development^14,15^ as well as for understanding molecular mechanisms in biochemistry and biophysics^16–19^. Often, the introduction of labels is simply impossible in such measurements, and the application of label-free methods is the only reliable choice. Unique advantages of label-free techniques are that they provide real-time kinetic information about molecular binding events and make the kinetic analysis possible even for low molar mass analytes. In this regard, the label-free principle has a crucial role in minimizing the interference in the measured tiny interactions. Using labels would strongly interfere with the binding and thus would not be able to provide reliable quantities on the system. Although the analysis of small molecules with 200–1000 Da molar mass is well established^20–22^, detection of analytes, especially characterizing their binding kinetics, with molar mass below 100 Da is still a great challenge. Importantly, most metal ions with significant biochemical role (e.g., Ca, Mg, Fe, Cu, Zn) or relevance in environmental samples (e.g., Ni, Cd, Hg, Pb) fall into this mass range. While the widely used surface plasmon resonance (SPR) biosensor has been already used for metal ion detection^23–27^, examples for studies measuring below 100 Da are especially rare and the binding kinetics has not been measured or analyzed in detail.

Recently, novel optical techniques have been developed to achieve sensitivity capable of recording binding kinetics for < 100 Da analytes. In this challenge, interferometry-based detection methods have proven to be particularly promising candidates also showing great potential for applications in point-of-care and integrated lab-on-a-chip devices^28–30^. Most interferometric techniques employ optical waveguides, above which the adsorption of molecules induces refractive index changes in a surface bound evanescent wave. Silicon technology is used to fabricate the sensor chips and usually one or multiple Y-shaped waveguides are integrated. The most commonly used measurement geometry is the Mach–Zehnder or Young interferometer configurations^31,32^. However, these setups suffer from disadvantages like alignment difficulties and high costs of components. The integration of the Young interferometers to parallel readout design is rather complicated. In the case of Mach–Zehnder interferometers the chip fabrication is high-priced and the alignment of the single mode channel with coupling fibers is difficult. Using dual polarization interferometry (DPI), it has been shown that highly sensitive interferometry-based biosensors might be capable of detecting the amount of ions binding to surface immobilized proteins^33,34^. Differential refractometer and DPI is sensitive to vibrations and not suitable for parallel readout^35^. The sensitivity of Young interferometers is 10^−7^ RIU (refractive index unit) in bulk solution and 1000 particles/mL for viruses. Mach–Zehnder interferometers have a sensitivity of 10^−7^ RIU in bulk solution and 20 pg/mm^2^ for proteins^36^.

**Figure 1:**
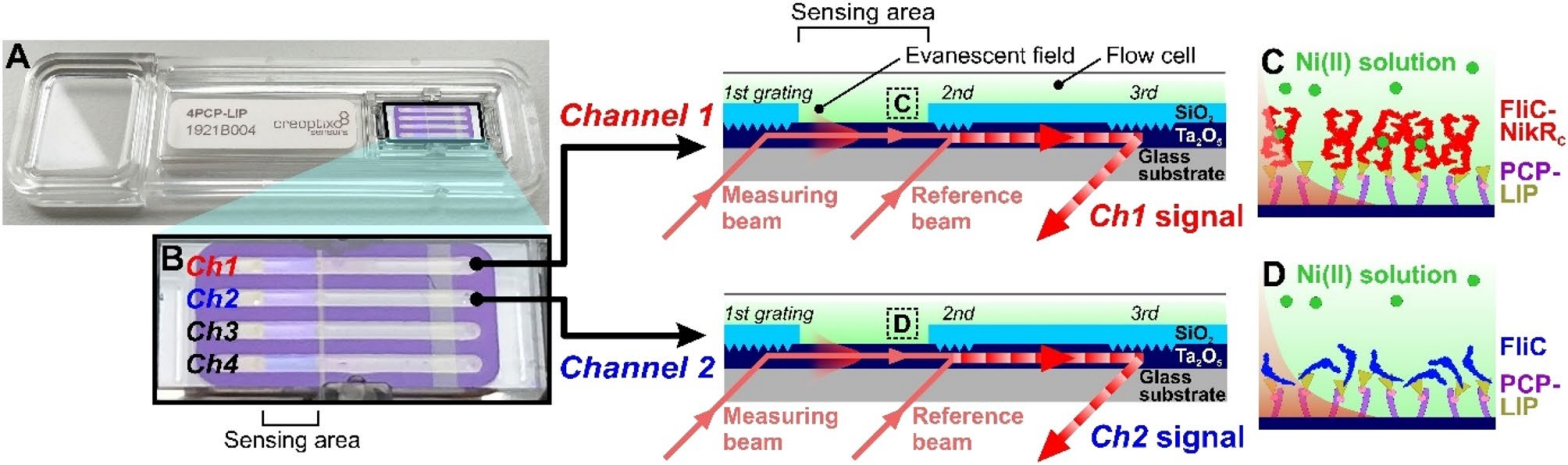
Structure of the applied 4-channel WAVEchip. (**A**) Full image of a PCP-LIP WAVEchip with its fluidic channels enlarged in (**B**) Two channels, a measurement and reference channel (*Ch1* and *Ch2*) are further detailed. The interference signal (phase-shift) is produced by the combination of the measuring beam and phase modulated reference beam inside the Ta_2_O_5_ waveguide film. The two beams are coupled through two optical gratings and the combined beam is coupled out by a 3rd grating. All the gratings and the path of combined beam are covered by a thin SiO_2_ layer (light blue) in order to avoid sensing of any refractive index change outside the indicated sensing area. The evanescent field generated by the electromagnetic mode propagating in the waveguide film (dark blue) is also indicated. Both channels have the same structure and are coated with an identical PCP-LIP coating. FliC-NikR_C_ protein was immobilized on *Ch1* and FliC on *Ch2*, respectively, and the same Ni(II) solution was simultaneously injected into the flow cell of both channels (**C**,**D**).

The recently developed grating-coupled interferometry (GCI) is a hybrid phase-shifting Mach–Zehnder interferometer, which can present several advantages over other optical biosensor techniques. It employs a simple waveguide-grating structure, moving parts are completely eliminated decreasing noise levels, the incorporated coupling gratings possess high alignment tolerance, and the applied Ta_2_O_5_ waveguide has high refractive index contrast with a relatively long interaction path with the sample. Moreover, unlike most interferometric setups employing spatial interference patterns, the interference appears in the time-domain for GCI. All of these features lead to outstanding optical stability, detection sensitivity and overall performances^35,37,38^, making GCI one of the most sensitive label-free biosensor currently available on the market. The GCI technology is capable of providing reliable kinetics even below signals of 1 pg/mm^2^ and it can be applied to determine the kinetic rate parameters of binding events with high sensitivity. This performance makes the technology suitable for characterizing the dynamic metal binding properties of surface-attached molecules. The structure of a GCI chip and its schematic working principle is shown in Fig. 1. It is important to stress that GCI with integrated precision fluid handling and temperature control is a well-established technique already applied in a number of fields, such as plant biology^18,19,39–44^, drug discovery^45^, natural products research^17^ cancer research^46^ as well as, most recently, exploring the receptor-binding domain of SARS-CoV2^47^. While GCI has already shown its potential in the binding kinetics measurement of a small polyphenol molecule, the 458 Da epigallocatechin gallate (EGCG)^17^, too, there are no examples in the scientific literature of exploiting its top-level sensitivity below molar mass of 60 Da. For demonstrating the performance of the GCI technology to study the binding kinetics of ions, we selected a newly developed genetically modified flagellin variant with Ni-binding ability, which can be potentially applied in further bio- and chemical sensor applications.

The bacterial flagellar filaments consist of thousands of flagellin subunits. Flagellin is composed of 4 domains: D0, D1, D2, and D3. The terminal D0 and partly D1 domains are disordered in the monomeric form^48^. These disordered regions are expected to easily adapt to the local environment. The hypervariable D3 domain is a largely independent part of flagellin, thus it can be removed or replaced by other proteins without disturbing the other domains of the molecule^49,50^. We have recently shown that flagellin or flagellin-based fusion proteins rapidly form an oriented, dense and stable monolayer on hydrophobic surfaces, where the hypervariable D3 domain is oriented towards the liquid phase^51,52^.

NikR is a metal-responsive transcription factor that controls Ni(II) uptake in *E. coli* by negatively regulating expression of a Ni-specific ABC transporter complex encoded by the *nikABCDE* operon. In the presence of excess intracellular Ni(II), the conformational change of the C-terminal Ni(II)-binding domain of the Ni-dependent repressor protein NikR (residues 49–133) enables the N-terminal domain (residues 1–48) to release the operator sequence of *nikABCDE,* and thus expression of the regulator proteins can begin^18^. Based on its structure, two of the C-terminal regulatory domains of *E. coli* NikR form a core dimer, which binds one Ni(II) per monomer with very high affinity^53^. Coordinating ligands come from both protein chains of a core dimer (H87, H89 and C95 from one, H76 from the other NikR subunit; Carrington et al.^54^), forming a square planar Ni(II) coordination sphere. Besides the high-affinity (HA) site, Ni(II) may bind to several other low-affinity (LA) binding sites per monomer^55^. For the HA site, dissociation constants (*K*_d_) in the picomolar/nanomolar range have been reported^56,57^, while the LA sites exhibit binding affinity in the micromolar range. The NikR C domain (NikR_C_) was demonstrated to form stable tetramers in solution by assembly of two core dimers. Ni-binding is not required for tetramerization^56^.

In this work, we have prepared a newly constructed FliC-NikR_C_ flagellin variant with inserting the C-terminal Ni-binding domain of NikR into the variable middle domain of FliC. With the development of this fusion protein, our aim was to produce a HA Ni-binding receptor for demonstrating the performance of the novel GCI technology. This interferometry-based biosensor method allowed for obtaining high-resolution kinetic data on the binding of the 59 Da Ni(II) analyte, whose kinetic detection is highly challenging even for today’s top biosensor technologies. The fully resolved kinetic data enabled us to determine the rate parameters and dissociation constants of the binding events even at very low immobilization levels. Besides the biosensor experiments, complementary isothermal titration calorimetry (ITC) measurements were also performed. Calorimetry is a widely used reference technique of label-free measurements (mainly shown for SPR in previous works^58–61^).

## Materials and methods

### Plasmid construction for NikR C-domain displaying flagellin variant

As a vector DNA, a modified pET19b (Novagen, Madison, Wisconsin, US) based plasmid was used which contains the gene of the D3-domain deficient flagellin protein between the NcoI and BamHI sites, excluding the N-terminal 10His coding sequence. In this plasmid the coding sequence of the D3 domain was substituted with a multiple cloning site (MCS) including four sequential restriction cleavage sites (XhoI, AgeI, XmaI and SacI) permitting the optimal incorporation of a peptide or protein coding sequence. Salmonella codon optimized gene of the C-terminal domain of *E. coli* NikR repressor protein (PDB ID: 3BKU) was synthesized and provided in between the EcoRV sites of a pUC57 vector by GenScript (Piscataway, New Jersey, US). Using the primers (forward) 5′-ACATACCGGTACCCAGGGCTTTGCGGTGC-3′ and (reverse) 5′-ACATGAGCTCATCTTCTTTCGGCAGGCACTGC-3′ *nikR* was amplified by PCR and placed into the modified pET19b plasmid using the AgeI and SacI sites (*fliC-nikR(C)*). As a result, the gene of a flagellin variant with the C-domain of NikR in it linked by the N-terminal ‘LETG’ and C-terminal ‘EL’ peptides was obtained. After the appropriate construction was confirmed by DNA sequencing, BL21 (DE3) cells were transformed by the pET19b plasmid containing *fliC-nikR(C)*.

### Protein expression and purification

20 mL LB media (Scharlau, Barcelona, Spain) with 100 µg/L ampicillin (PanReac AppliChem, Darmstadt, Germany) (LB/Amp) was inoculated from freshly prepared LB/Amp agar plate of BL21 (DE3) bacteria containing the FliC-NikR_C_/pET19b vector, grown at 37 °C with 270 rpm shaking until optical density at 600 nm (OD_600_) was between 0.6 and 1.0 and kept at 4 °C. Next morning 5 mL of the starter media was added to 1 L LB/Amp in a 3 L volume baffled flask and grown at 37 °C with moderate shaking (100 rpm). When OD_600_ was between 0.4 and 0.6, culture was induced with 0.5 mM isopropyl β-D-1-thiogalactopyranoside (IPTG, Sigma-Aldrich, St. Louis, Missouri, US), and further incubated at 30 °C for 4 h. After that, cells were harvested by centrifugation (4300×*g*, 30 min, 10 °C on a Heraeus Multifuge 3 S-R, Heraeus, Hanau, Germany), washed with 20 mM Na_2_HPO_4_, 150 mM NaCl, pH 7.8 buffer, centrifuged and stored at - 80 °C. Applying the above process, typically 4–5 mL cell pellet was given from 1 L LB media. During the whole purification process cells were kept on ice. The cell pellet from 1 L growing was resuspended in 20 mL 20 mM Na_2_HPO_4_, 150 mM NaCl, pH 7.8 buffer with 2 Complete Mini EDTA-free protease inhibitor tablets (Hoffmann-La Roche, Basel, Switzerland) and lysed by sonication using 50% intensity, 5 times 1 min, cooling in between on an Omni Sonic Ruptor 400 instrument (Omni Instruments Ltd., Dundee, UK). The lysate was centrifuged with 82,000×*g* for 25 min on an Optima Max-E Ultracentrifuge (Beckman Coulter, Brea, CA) and the supernatant was filtered by a 0.22 µm sterile syringe filter. Imidazole in 10 mM concentration was added to the solution prior to Ni(II)-affinity chromatography to prevent non-specific binding to the column. The FliC-NikR_C_ containing solution was loaded onto a 5 mL HiTrap Chelating (GE Healthcare, Chicago, Illionis, US) column, previously equilibrated by buffer A (100 mM K-phosphate, 500 mM NaCl, 10 mM imidazole, pH 8.0). After washing the column with buffer A, the bound protein was eluted by buffer B (100 mM potassium phosphate, 10 mM Tris, 250 mM imidazole, pH 7.6) into EDTA solution (pH 8.0, final concentration is 10 mM) to avoid dimer or tetramer formation or aggregation derived by metal ion coordination.

### Isothermal titration calorimetry

ITC measurements were performed to characterize the Ni-binding properties of the FliC-NikR_C_ fusion protein, using a VP-ITC instrument (MicroCal, Northampton, MA, USA). For calorimetric measurements buffer in FliC-NikR_C_ protein solution was first exchanged by 20 mM 4-(2-hydroxyethyl)-1-piperazineethanesulfonic acid (HEPES), 150 mM NaCl, pH 7.0 using a 5 mL HiTrap Desalting (GE Healthcare) column, and the collected fraction was further dialyzed overnight against the same buffer. An approximately 30 mM stock solution of NiSO_4_ was prepared by dissolving the solid salt in the overnight dialysis buffer of the protein, equilibrated at room temperature for an hour to ensure identical buffering conditions for the measurements. The sample cell was loaded with a 90 µM protein solution and it was titrated by a 1.8 mM NiSO_4_ solution. ITC experiments were performed at 25 °C with 7 µL injection volumes and 240 s equilibration times between injections. As a control experiment, the NiSO_4_ was also injected into the buffer, and the measured heats of dilution were subtracted from the main experiment. Calorimetric data were analyzed using MicroCal Origin software fitting them by a sequential binding sites model, assuming 3 binding sites.

### Grating-coupled interferometry

The binding kinetics of Ni(II) on FliC-NikR_C_ was measured by the label-free, high sensitivity GCI WAVE instrument (Creoptix AG, Wädenswil, Switzerland)^35,38^. In the present study, both the two-channel (WAVE) and the next-generation four-channel (WAVEdelta) instruments were used. For simplicity, we hereafter refer to the technique as WAVE in general. The experiments were conducted employing specific optical sensor chips, the so-called WAVEchips (Creoptix AG) (see Fig. 1). In order to analyze the effect of immobilized flagellin amount on Ni detection, two types of WAVEchips with different functional coatings, PCP-LIP and PCH were applied. PCP-LIP refers to a thin, quasi-planar polycarboxylate polymer (PCP) coating on the waveguide where lipophilic (LIP) groups are pre-immobilized to the PCP chains. This type of sensor chip can be used for capturing ligands through hydrophobic interactions (according to our previous results, flagellins can be efficiently captured in this way^52,62,63^). The PCH chip carries a thick polycarboxylate-based hydrogel coating with free carboxylic functions to which proteins can be covalently coupled through EDC/NHS (1-ethyl-3-(3-dimethylaminopropyl)carbodiimide hydrochloride/N-hydroxysuccinimide) linking chemistry.

Owing to their multiple-channel design, WAVEchips allow for referencing the signal recorded on the target channel with a signal measured on a selected reference channel. In our experimental design, we immobilized Ni-responsive FliC-NikR_C_ to the target channel (*Ch1*), while Ni-insensitive FliC was captured on the reference channel (*Ch2*). The handling of all the different solutions including sample pickup, delivery and injection onto the channels was performed fully automatically by a robotic autosampler (WAVEsampler).

Throughout the entire experimental period 10 mM HEPES, 150 mM NaCl, pH 7.4 was used as running buffer (RB). The basic element of a GCI experiment is the cycle, which consists of a baseline (RB injection), a sample (e.g., protein solution) injection as well as a washing section (RB injection). A typical GCI experiment can be separated into a sequence of series, where a series is usually composed of more cycles. As a first experimental step, the polycarboxylate layer of the WAVEchip was conditioned using a solution of 0.1 M sodium tetraborate decahydrate (borate; VWR, Hungary), 1 M NaCl, pH 9.0, which was followed by sequential running buffer injections (so-called startup cycles). In the case of PCP-LIP chips, the flagellin molecules were immobilized to the surface by physisorption on the lipophilic anchoring groups. While 1 mg/mL FliC-NikR_C_ solution (dissolved in the RB) was injected into *Ch1*, 1 mg/mL FliC solution also prepared in RB was injected into *Ch2* (the preparation of monomeric FliC solution from filaments was described elsewhere^52^). The experimental conditions of the covalent immobilization of FliC and FliC-NikRC on PCH chip can be found in the Supplementary Information: Note 1. After the immobilized protein layer was stabilized with sequential RB flows (stabilizing startup cycles), dilution series of NiSO_4_ solutions (prepared in RB) covering the concentration region of 1 nM–10 μM were simultaneously injected both into channels *Ch1* and *Ch2*. For double referencing, we applied Ni(II)-free RB injections (blanks) at every 5th Ni(II) cycle.

The control of the device as well as data adjustment and kinetic analysis were all performed using the WAVEcontrol software (Creoptix AG). The raw kinetic data were adjusted using X,Y-offset and blank correction and the processed data were fitted using the “heterogeneous ligand” kinetic model of the WAVEcontrol software (for the model equations, see Supplementary Information: Note 2).

## Results and discussion

### Construction, bacterial production and purification of FliC-NikR_C_

In order to replace the D3 domain of flagellin with NikR_C_, it is essential to use appropriate linker segments which allow proper folding of both fusion partners. Selection of N- and C-terminal linker peptides with appropriate length for the incorporation of the C domain of NikR into flagellin was done by computer modelling based on the structure of *E. coli* NikR C-domain (PDB ID: 3BKU) and *S. typhimurium* flagellin (PDB ID: 1UCU). Since the N- and C-terminal ends of NikR_C_ are closely separated by only about 6 Å, we concluded that short linkers at both ends of NikR_C_ would be sufficient. Linker peptides with 2, 4 or 6 amino-acid-length are encoded in our pre-fabricated pBR322 based plasmid DNA (NT045) containing the D3-domain deficient flagellin gene^64^. We chose peptides LETG and EL as linkers at the N- and C-terminus of NikR_C_, respectively. To generate these linker peptides, the *nikR*_*C*_ gene was incorporated into NT045 by using the AgeI and SacI restriction sites.

Flagellin-based fusion proteins may form flagellar filaments on the cell surface making protein purification simple and cost effective. Expression of FliC-NikR_C_ was first attempted in flagellin deficient SJW2536 *S. typhimurium* cells, but secretion of the fusion protein was not observed. Therefore, for efficient protein production the *fliC-nikR*_*C*_ gene was introduced into a modified pET19b vector, expressed in *E. coli* BL21 (DE3) strain, and the FliC-NikR_C_ fusion protein was purified by Ni-affinity chromatography. Our optimized protein expression protocol resulted in about 35 mg soluble protein per 1 L LB medium.

**Figure 2:**
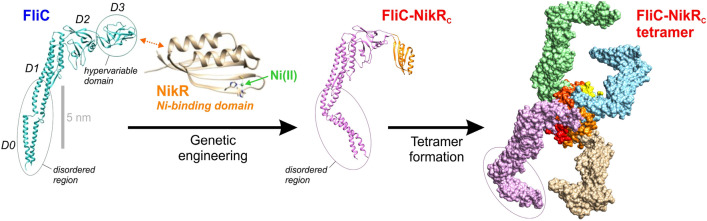
The 3D structure of the wild-type flagellin FliC, the genetically engineered FliC-NikR_C_ and the FliC-NikR_C_ tetramer that is spontaneously formed in solution. Each monomer unit of the FliC-NikR_C_ tetramer has one HA Ni-binding site. Note that the D0 and partly the D1 domain forms a disordered region displaying high flexibility. While the structure of FliC (PDB ID: 1UCU) and NikR C-domain (PDB ID: 3BKU) is well established by electron microscopy, the shown structure of monomeric and tetrameric FliC-NikR_C_ is based on modeling using the Chimera Software^65^. Due to the conformational variability in its disordered arms, only a hypothetical structure could be shown for the FliC-NikR_C_ tetramer.

*E.coli* NikR or its C-terminal domain is known to form tetramers in solution. To characterize the oligomerization state of the purified FliC-NikR_C_ sample dynamic light scattering (DLS) measurements were performed. These experiments revealed that FliC-NikR_C_ forms tetrameric assemblies similarly to NikR_C_^71^ (for DLS results, see Fig. S1 in the SI). The modeled structure of FliC-NikR_C_ is shown in Fig. 2.

### ITC study of Ni-binding by FliC-NikR_C_

Ni-binding properties of the FliC-NikR_C_ construct were characterized in solution by ITC. Measurements were done in non-complexing HEPES buffer which is ideal for Ni-binding studies. Previous works suggested that NikR_C_ contains two distinct classes of Ni-binding sites with largely different affinities^49^. Calorimetric measurements were applied to demonstrate that NikR_C_ preserved its Ni-binding ability upon its internal fusion into flagellin.

The obtained calorimetric profile shows a multistep binding process and a complex behavior at low Ni concentrations. The ITC data indicate the presence of a tight binding process finished after an equimolar Ni(II) added per FliC-NikR_C_ monomer. This process presumably reflects interaction of Ni ions with the HA sites of NikR_C_. While the HA site was being filled, a parallel endothermic event followed the fast initial exothermic metal binding step. Spontaneous endothermic processes are driven by the increase of entropy, which may be caused by the release of water molecules from the hydration sphere of the protein, due to either conformational changes and/or protein oligomerization. The latter possibility was not supported by DLS measurements performed at various Ni(II) concentrations which demonstrated that the oligomeric state of the FliC-NikR_C_ fusion protein was not significantly influenced by the presence of Ni ions (data not shown).

A very similar calorimetric behavior was observed by Zambelli et al.^66^ upon titration of *H. pylori* NikR_C_ with Ni(II), who concluded that the endothermic calorimetric component reflects internal conformational rearrangements induced by Ni(II) binding to the HA sites. It seems that a similar process occurs in FliC-NikR_C_, but after addition of half Ni(II) equivalent this process was completed and the endothermic component disappeared.

**Figure 3:**
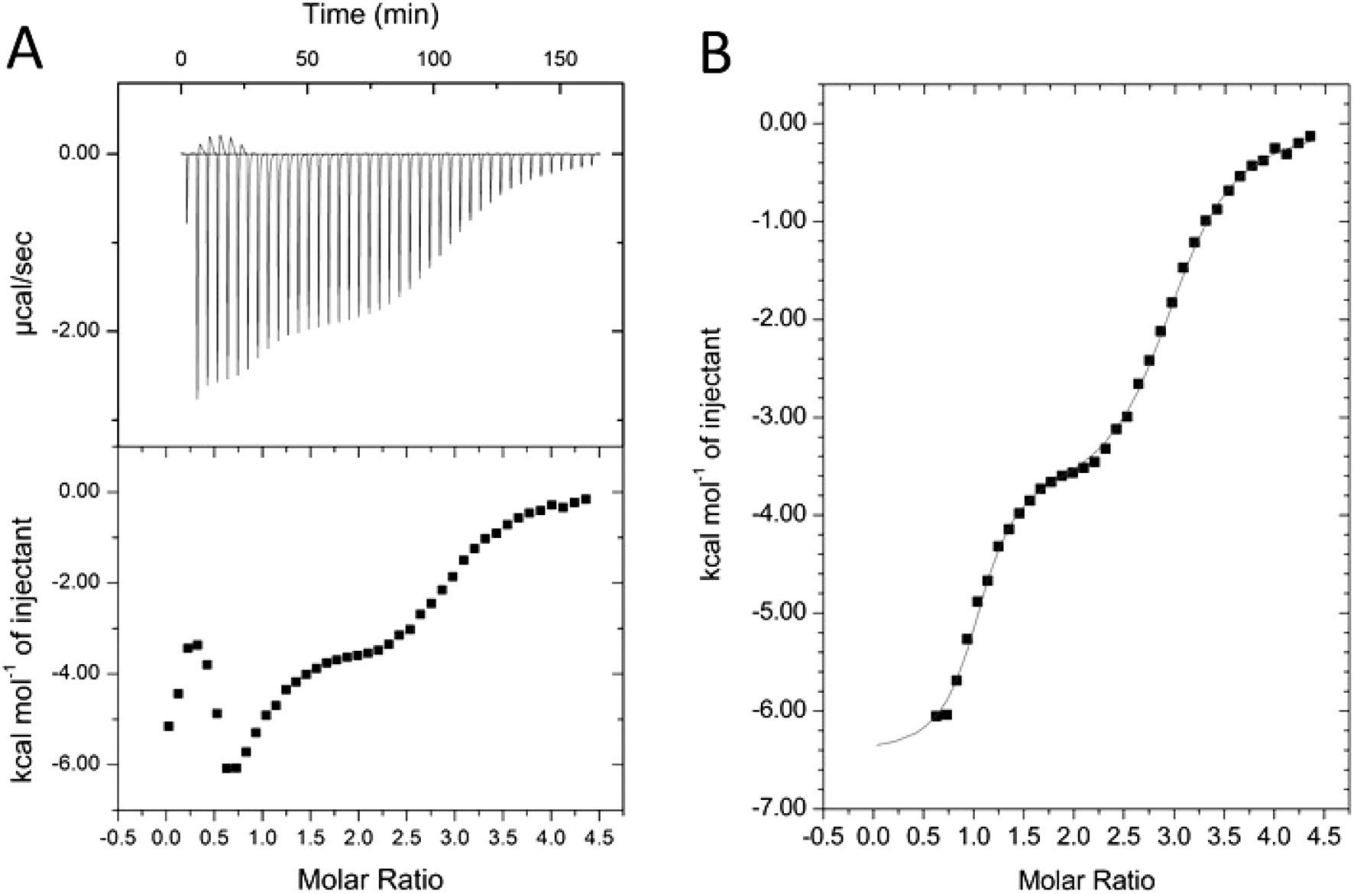
ITC results on the determination of the *K*_d_ values of FliC-NikR_C_ binding sites in solution. (**A**) Raw (top) and integrated (bottom) thermograms. (**B**) The measured data points were fitted by the “Sequential Binding Sites” model (MicroCal) assuming 3 Ni-binding sites.

The very first portion of the calorimetric profile was too complex for evaluation. The first few data points influenced by the endothermic after-peaks were excluded from the analysis, and the remaining portion of the titration curve was attempted to fit by various binding schemes. Good fit was only obtained by a three sequential binding site model. This model is a reasonable approximation also for independent binding sites with largely different affinities. As shown in Fig. 3, the measured data were nicely fitted with the applied model with binding parameters summarized in Table 1. For the HA binding a *K*_d_ of ca. 60 nM was obtained, while binding of two additional Ni(II) ions at LA sites occurred with a *K*_d_ of 2.1 and 8.9 µM, respectively. The LA sites are characterized by rather similar thermodynamic parameters. However, the values obtained for Δ*H* and Δ*S* are apparent, and include contributions not only from Ni(II) binding, but also from associated events such as deprotonation of the cysteines and consequent change in the buffer ionization state.

**Table 1:** Thermodynamic parameters for the best fit of ITC binding curve by the “Sequential Binding Sites” model.

	*K*_d_ (M)	Δ*H* (cal/mol)	Δ*S* (cal/mol/deg)
HA binding site	62.5 × 10^–9^	− 6301	11.8
LA binding site 1	2.13 × 10^–6^	− 3897	12.9
LA binding site 2	8.93 × 10^–6^	− 3731	10.6

In summary, our results suggest that FliC-NikR_C_ binds Ni(II) ion at a HA site with dissociation constant in the 10^−8^ M range, and contains two additional LA binding sites with a *K*_d_ values in the µM region.

### Ni binding kinetics revealed by grating-coupled interferometry

We analyzed the binding kinetics of Ni(II) ions to FliC-NikR_C_ using GCI under various conditions including different types of sensor surfaces for flagellin immobilization as well as low and high coverages of FliC-NikR_C_.

**Figure 4:**
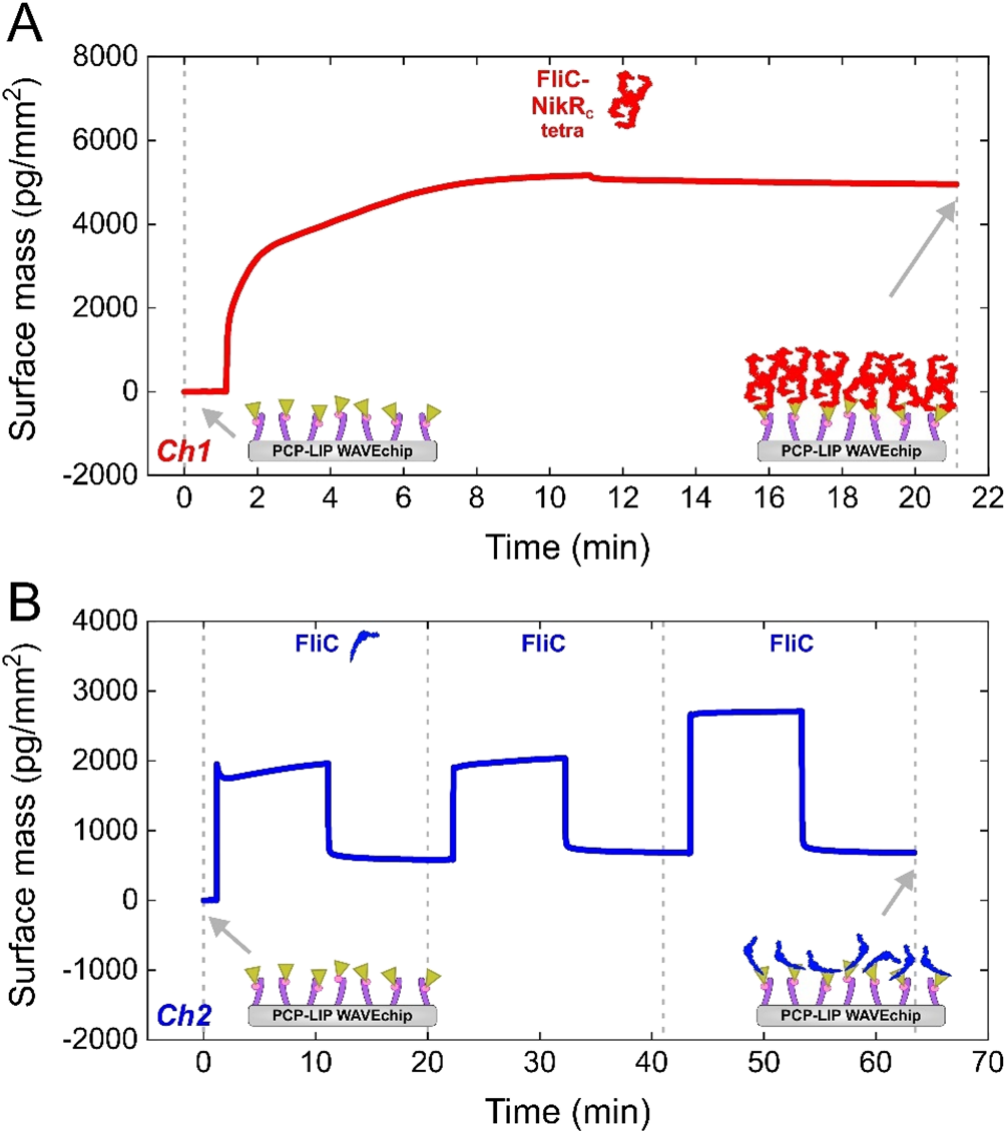
(**A**,**B**) FliC-NikR_C_ and FliC immobilization series performed on PCH chip using channel *Ch1* and *Ch2*, respectively. The series included the injection of FliC-NikR_C_ and FliC solution reaching 4950 pg/mm^2^ and 680 pg/mm^2^ adsorbed masses, respectively. All cycles involve a baseline (RB injection), a flagellin injection as well as a washing (RB injection) section (cycle sections are not indicated on the graph but can be followed according to the upgoing/dropping curves). The schemes on the bottom of the graphs illustrate the quasi-planar PCP coating with lipophilic functions and immobilized flagellin molecules.

Figure 4A,B represents the immobilization of FliC-NikR_C_ and FliC to the two separate channels of the lipophilic PCP-LIP chip, which has a quasi-planar PCP coating with lipid anchoring motifs. The detected much higher amount of deposited mass of FliC-NikR_C_ as compared to FliC (4950 vs. 680 pg/mm^2^) can be attributed to the higher molecular weight and larger interaction surface of the tetrameric FliC-NikR_C_. Assuming monolayer coverage and considering the molecular weight of flagellins (FliC-NikR_C_ tetramer: 207.556 kDa, FliC: 51.612 kDa), from the measured surface mass one can calculate the effective occupied area of a single protein molecule. These areas were found to be 70 and 126 nm^2^ for FliC-NikR_C_ tetramer and FliC, respectively. Taking into account that a single FliC-NikR_C_ and FliC molecule attached to the surface through its hydrophobic D0 domain(s) has an average realistic footprint of around 30 and 20 nm^2^, the average separation distance between the immobilized molecules is 2.9 and 6.8 nm, respectively. However, the different shape and magnitude of the kinetic curves in Fig. 4A,B suggests that the tetrameric FliC-NikR_C_ presumably interacts with the chip surface with multiple bonds, leading to an almost irreversibly adsorbed layer. While in case of FliC, a large amount of the molecules adsorbs reversibly and can be easily washed off, suggesting a reversible and an irreversible adsorption form.

**Figure 5:**
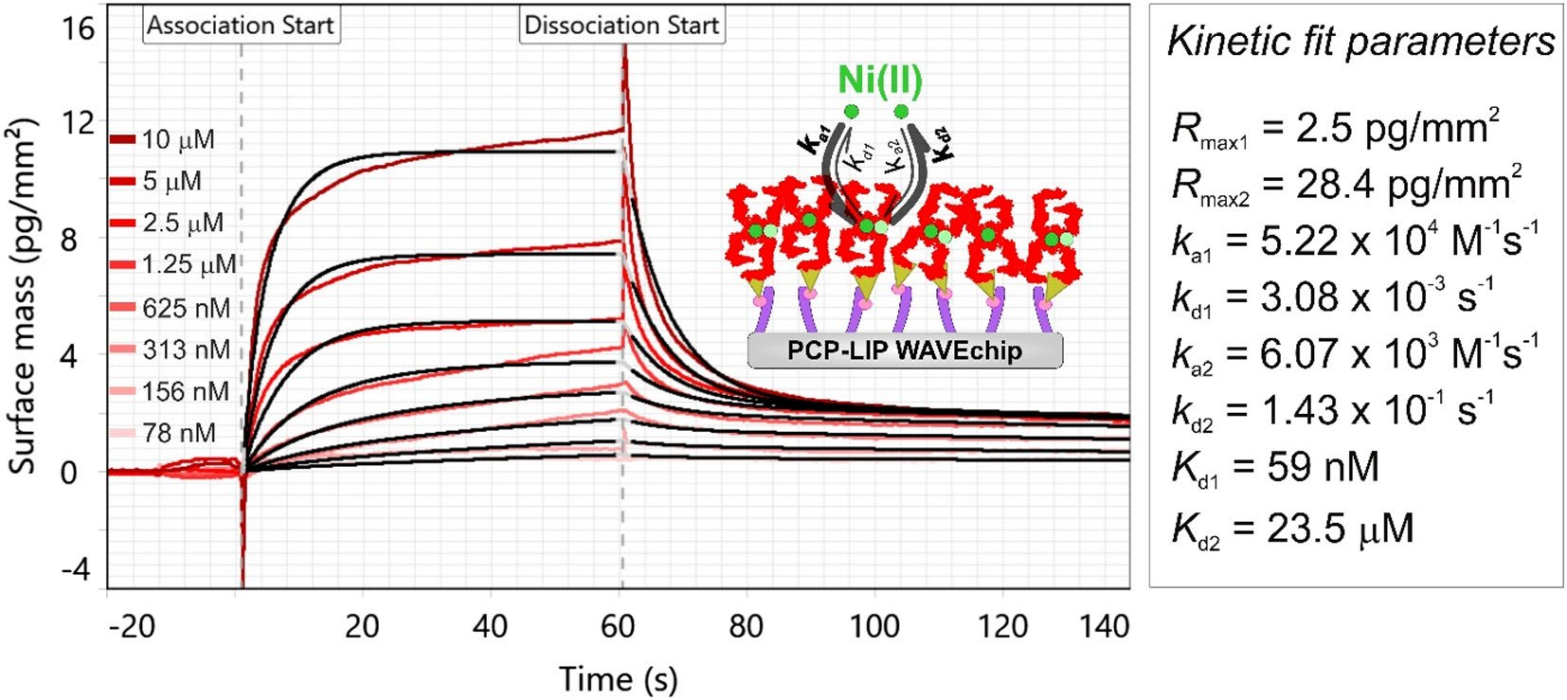
Measured kinetic data of Ni(II) binding obtained at high FliC-NikR_C_ coverage on a PCP-LIP WAVEchip (red curves). The shown measurement curves were adjusted and represent *Ch1*-*Ch2* reference corrected data. The data were fitted using the heterogeneous ligand kinetic model (black curves). The spikes at association start and dissociation start annotations were originated from solution exchange effect. The inset scheme illustrates the binding of Ni to the FliC-NikR_C_ tetramers which were immobilized on the quasi-planar lipophilic coating of the PCP-LIP chip. While the solid circles with dark green color represent Ni(II) ions bound to the HA sites, the light green circles represent Ni(II) ions bound to the LA sites. Kinetic data determined from the model fit are shown in the right table. Here, subscript index 1 and 2 correspond to the HA and LA sites, respectively, *R*_max_ is the maximal response value, *k*_a_ and *k*_d_ are the association and dissociation rate constants as well as *K*_d_ is the dissociation constant.

Figure 5 shows the measured kinetic data evaluated by the heterogeneous ligand kinetic model and kinetic parameters resulted from the model fit are also presented next to the graph. While other models (called as “1: 1 kinetic” and “bivalent analyte” model in the WAVEcontrol software) were also tested to fit the data, as a confirmation of the expectations based on ITC measurements, the best fit quality could be achieved by the heterogeneous ligand model. According to the results, two types of binding sites with different affinities were detected: one with a higher dissociation constant, meaning low (*K*_d1_ = 23.5 µM) and one with a lower dissociation constant, meaning high Ni(II)-binding affinity (*K*_d2_ = 59 nM). The fact that the model with two types of Ni-bindings sites on FliC-NikR_C_ provided the best fit and that the determined *K*_d_ values of those sites agreed well with calorimetric data, strongly confirms our results and the performance of the GCI method.

Based on the *R*_max_ values obtained for the HA and LA sites (2.5 vs. 28.4 pg/mm^2^), we found that the proportion of the HA and LA sites on a FliC-NikR_C_ monomer is about 1:11. The finding that there are significantly more LA binding sites is well supported by observations of previous studies. According to the literature, this number can vary in the range of 12–32 per tetramer^55,57,67^. It is obvious from this large deviation that there is no clear consensus in the number of LA sites, which can be attributed to the difficulty of differentiating between them and determine their exact number. Considering the fact that one FliC-NikR_C_ tetramer has 4 HA sites, our results suggest approximately 44 LA sites on one tetramer.

**Figure 6:**
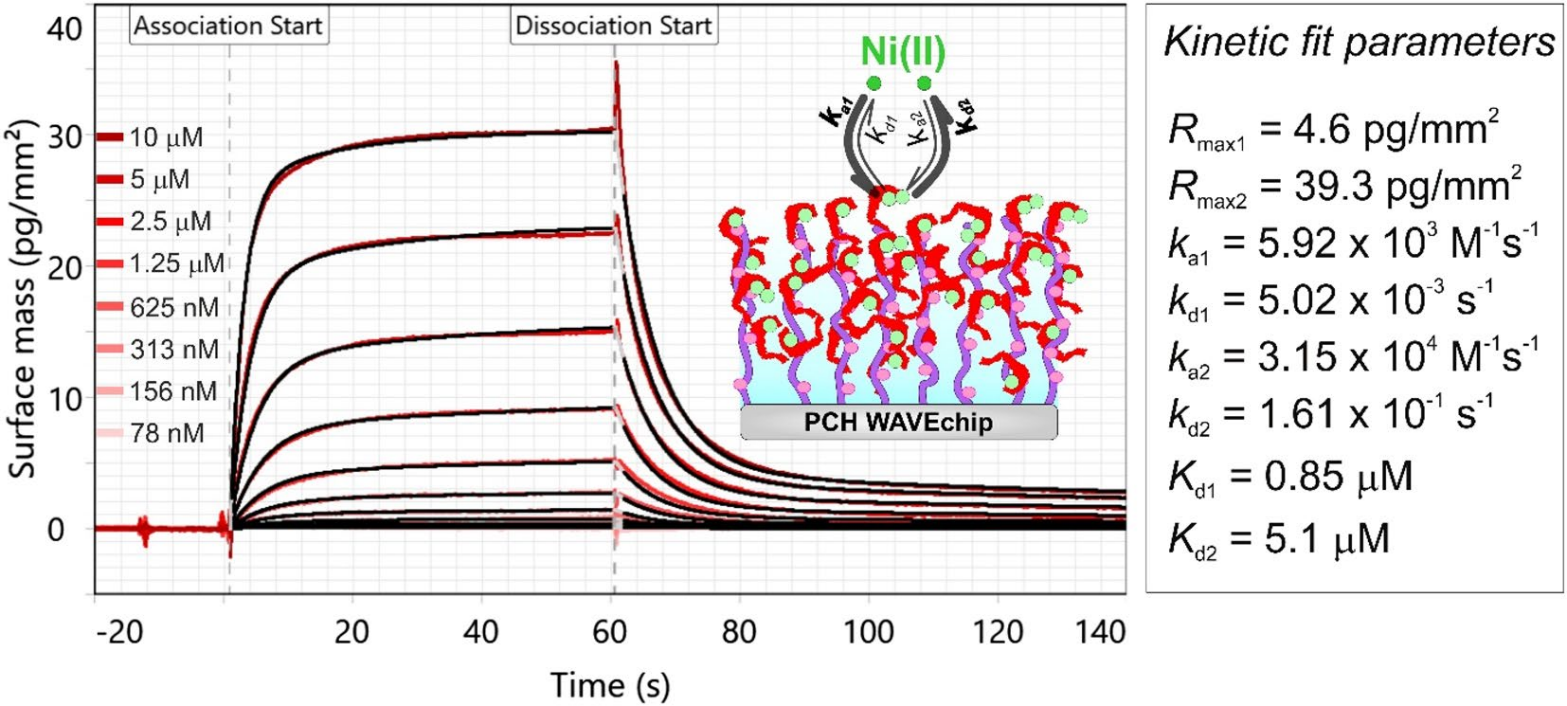
Measured kinetic data of Ni(II) binding obtained at high FliC-NikR_C_ coverage on a PCH WAVEchip (red curves). The shown measurement curves were adjusted and represent *Ch1*-*Ch2* reference corrected data. The data were fitted using the heterogeneous ligand kinetic model (black curves). The inset scheme illustrates the binding of Ni to the FliC-NikR_C_ monomers which were immobilized on the thick hydrogel coating of the PCH chip. The light green circles represent Ni(II) ions bound to LA sites.

Besides the physisorption of flagellin onto the PCP-LIP surfaces, we also applied covalent immobilization to the hydrogel-based PCH chip that carries a high-capacity 3D immobilization matrix with carboxyl groups. The details and results of these experiments on covalent immobilization are detailed in the Supplementary Information: Note 4. Whilst significant difference between the two channel signals could be clearly detected on the PCH surface too, the kinetic analysis reflected that only one type of Ni(II) binding site exists with a *K*_d_ of 1–6 µM (Fig. 6). Our result that the HA site disappears can be explained by the applied acidic pH at which the immobilization was performed. This finding is well supported by the observation of Fauquant and co-workers^55^ that the tetrameric structure of FliC-NikR_C_ breaks up at pH below 5, leading to the loss of HA sites.

**Figure 7:**
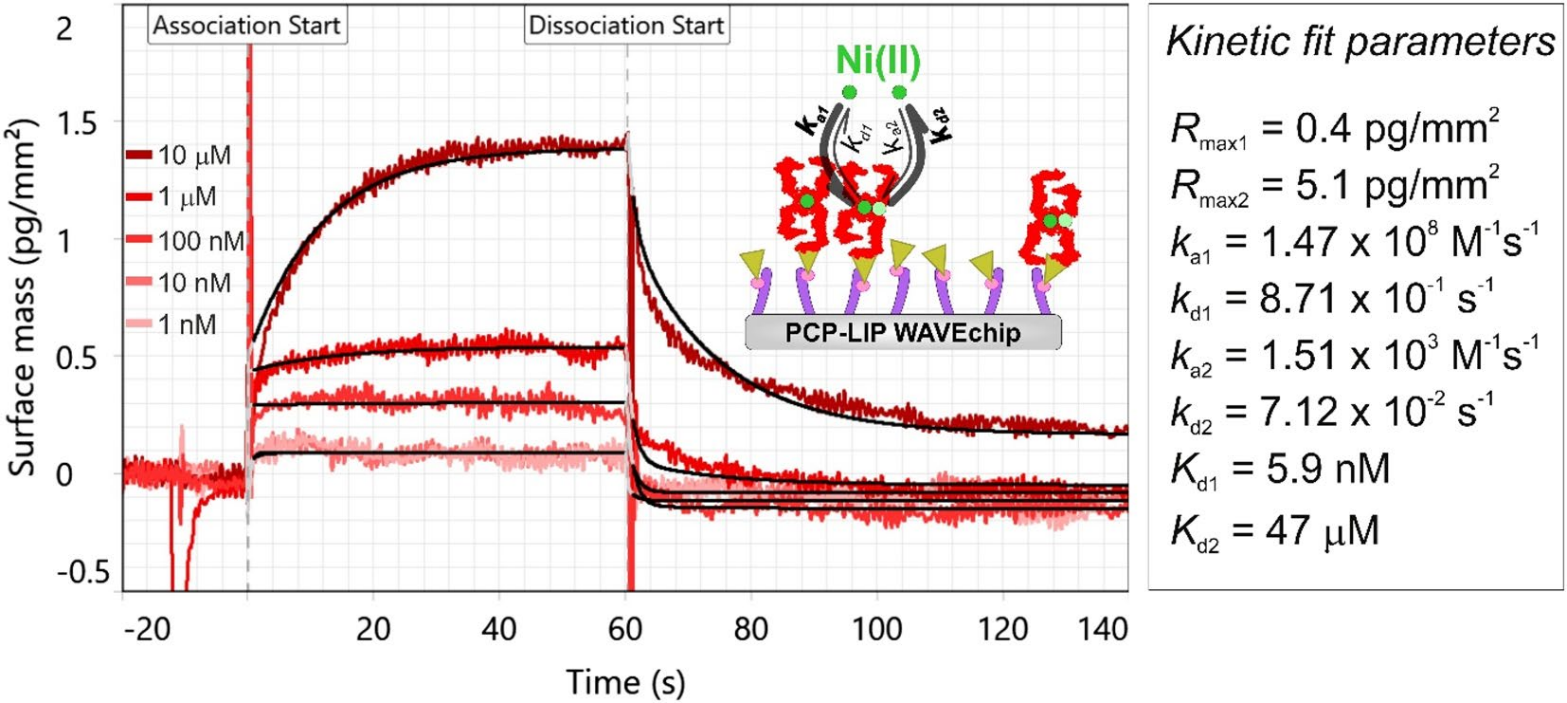
Measured kinetic data of Ni(II) binding obtained at low FliC-NikR_C_ coverage on a PCP-LIP WAVEchip (red curves). The shown measurement curves were adjusted and represent *Ch1*-*Ch2* reference corrected data. The data were fitted using the heterogeneous ligand kinetic model (black curves). The inset scheme illustrates the binding of Ni to the FliC-NikR_C_ tetramers which were immobilized on the quasi-planar lipophilic surface of the PCP-LIP chip. While the solid circles with dark green color represent Ni(II) ions bound to the HA sites, the light green circles represent Ni(II) ions bound to the LA sites.

To push the limits of the GCI technology, we also measured the Ni(II)-binding kinetics of FliC-NikR_C_ when a much lower amount of protein tetramers was immobilized on the PCP-LIP surface (surface mass 840 pg/mm^2^, separation distance 15 nm). Due to the low atomic weight of the Ni analyte and the low immobilization density, extremely low signals (0.1–1.4 pg/mm^2^) were obtained, which however, fell into the detectable range and as shown in Fig. 7, the kinetic curves corresponding to the separate concentrations could be efficiently resolved. The obtained *K*_d_ values are in reasonable agreement with the results obtained on PCP-LIP chip at high coverage. In addition, compared to the high coverage PCP-LIP measurement, the proportion of the *R*_max_ values corresponding to the HA and LA sites (1:13) could be also well reproduced. The fact that the kinetic curves could be resolved even at the 0.1–1.4 pg/mm^2^ range clearly demonstrates the unprecedented sensitivity of the GCI technology.

It is important to note that both the kinetic constants and the dissociation constant of the binding for the LA site is approximately the same, irrespective of the layer density of the tetramer molecules. However, interestingly, large deviations are seen for the HA binding sites. We believe that these discrepancies are in close connection with the deposition density of molecules and available space for the disordered terminal regions fixing the tetramers on the surface. It can be reasonably assumed that in case of low coverage, the tetramer is immobilized through 2–3 arms, affecting accessibility of the HA sites. Since the LA sites are not affected by the tetramer formation^56^, their binding properties remain approximately the same, independent of the immobilization density which is in full agreement with our observations. This finding shows that performing an affinity measurement at low and high immobilization densities can potentially reveal important properties of the system. This further highlights the need of high detection sensitivity. Of note, similar observations were reported previously. We would like to stress that it is well-demonstrated^68–71^ that the chip type, the properties of the surface layer and the way of immobilization generally affect the apparent binding affinity, resulting in a difference in the measured *K*_d_ values determined by ITC and label-free methods (like SPR). This effect is not special for GCI, it is true for other methods based on surface sensing layers. This is not surprising, since immobilization can strongly affect the local environment of the molecules and potentially introduces some steric constraints, too.

## Conclusions

In this study, we have demonstrated the unprecedented capability of the GCI label-free biosensor technique in detecting the interaction of extremely small < 100 Da analytes with their target ligand and analyzing the kinetics of the binding events.

The interaction examined in this work was the binding of Ni(II) ions (59 Da) to a newly developed FliC-NikR_C_ flagellin variant designed to possess Ni(II)-responsive property in the submicromolar range. For this purpose, part of the transcription factor NikR, which has Ni-binding sites, was incorporated into the wild-type FliC flagellin using genetic engineering.

The binding kinetics of Ni(II) was measured under various conditions including different chip surface chemistries used for flagellin immobilization (PCP-LIP, PCH) as well as varying the coverage level of immobilized FliC-NikR_C_ (low and high coverages). Summary of the Ni(II) kinetic data obtained by the GCI technique are summarized in Supplementary Information: Note 5. Based on the measurements on PCP-LIP lipid anchoring surfaces at high FliC-NikR_C_ coverage (4950 pg/mm^2^), we found that FliC-NikR_C_ has two types of Ni-binding sites which present different Ni affinities, involving low-affinity (*K*_d_ = 23.5 µM) and high-affinity (*K*_d_ = 59 nM) binding sites. This result is supported reasonably well with ITC data obtained in solution at equilibrium. Measurements carried out with covalently immobilized FliC-NikR_C_ on PCH surface confirmed that the HA binding site is sensitive to the pH: at acidic pH (around pH 3.5), the tetrameric structure of FliC-NikR_C_ breaks up, losing its HA sites. The application of different surface chemistries revealed that the dynamic behavior of HA binding sites is affected by the FliC-NikR_C_ immobilization density. Note, the immobilization affected the *K*_d_ and *k*_a_ value, but *k*_d_ was less influenced. Interestingly, we observed similar behavior in case of integrin receptors binding the RGD peptide motif in a locally varied environment due to enzymatic digestion of the glycocalyx around the target integrins (Kanyo et al. under publication). At low immobilization densities the need for high sensitivity is even more pronounced. Exploiting the top-level detection limit of the present method, we could detect the presence of nM binding sites even when the FliC-NikR_C_ was immobilized at low coverage level to the PCP-LIP surface (840 pg/mm^2^). This result proves that the interferometric detection technique with the applied double referencing method is fully capable of revealing the binding kinetics of a 59 Da metal ion even at low immobilized ligand level. These results could open up new possibilities in biology, especially in the research of metal-binding proteins.

## Supplementary information

Supplementary Information.
