# Supplementary material for: Grating-coupled interferometry reveals binding kinetics and affinities of Ni ions to genetically engineered protein layers
Source: Sci Rep. 2020 Dec 17;10:22253. doi: 10.1038/s41598-020-79226-w (PMC7746762; doi:10.1038/s41598-020-79226-w)
Supplement: Supplementary file 1 — Supplementary Information. [file 41598_2020_79226_MOESM1_ESM.docx]

**Grating-coupled interferometry reveals binding kinetics and affinities of Ni ions to genetically engineered protein layers**

Hajnalka Jankovics^1#^, Boglarka Kovacs^2#^, Andras Saftics^2^, Tamas Gerecsei^2^, Éva Tóth^1^, Inna Szekacs^2^, Ferenc Vonderviszt^1,2^, Robert Horvath^2*^

*^1^ Bio-Nanosystems Laboratory, Research Institute of Biomolecular and Chemical Engineering, University of Pannonia, Egyetem u. 10, Veszprém, Hungary*

*^2^ Nanobiosensorics Group, Institute of Technical Physics and Materials Science, Centre for Energy Research, 29-33 Konkoly-Thege Miklós út, Budapest, Hungary*

*^#^ These authors equally contributed to this work.*

*^*^* *Corresponding author (emails:* [*horvath.robert@energia.mta.hu*](mailto:horvath.robert@energia.mta.hu) *,* [*r74horvath@gmail.com*](mailto:r74horvath@gmail.com) *,* [*horvathr@mfa.kfki.hu*](mailto:horvathr@mfa.kfki.hu) *)*

***Note 1: Covalent immobilization on PCH chips: methodology***

In the case of experiments with PCH chips, the covalent immobilization of the flagellins was preceded by a pH scouting measurement series. The purpose of this experimental phase was to find the optimal pH at which FliC-NikR_C_ and FliC molecules could be adsorbed to the surface (closest pH from the RB showing sufficient surface density increase). The flagellin solutions (100 µg/mL) were prepared in 10 mM acetate buffers whose pH was set to 3.5, 4, 5 and 6. All the protein injection cycles were followed by a regeneration cycle involving the injection of 0.1 M borate, 1 M NaCl, pH 9.0. The optimal immobilization solution found during pH scouting was used in the subsequent covalent immobilization of flagellins. This series started with the activation of carboxyl groups using a 1 : 1 mixture of 0.4 M EDC and 0.1 M NHS aqueous solutions (both reagents were obtained from Sigma-Aldrich Chemie GmbH, Schelldorf, Germany), then 100 µg/mL FliC-NikR_C_ and FliC solutions prepared in 10 mM acetate buffer at the selected pH was injected into *Ch1* and *Ch2*, respectively. These cycles were followed by the passivation of the unreacted carboxyls using a 1 M ethanolamine (EA, Sigma-Aldrich Chemie GmbH, Schelldorf, Germany), pH 8.0 solution.

***Note 2: Fitted kinetic model***

The measured data were fitted using the “heterogeneous ligand” kinetic model of the WAVEcontrol software. According to the model, the binding process can be described with the following scheme:

*k_d1_*

*k_a1_*

*k_a2_*

*k_d2_*

| $L_{1}+A\rightleftharpoons L_{1}A$ | (1a) |
| --- | --- |
| $L_{2}+A\rightleftharpoons L_{2}A$ | (1b) |

and the corresponding rate equations can be written as follows:

| $\frac{d\left[ L_{1} \right]}{dt}=-(k_{a1}\left[ L_{1} \right]\left[ A \right]-k_{d1}\left[ L_{1}A \right])$ | (2a) |
| --- | --- |
| $\frac{d\left[ L_{1}A \right]}{dt}=\left( k_{a1}\left[ L_{1} \right]\left[ A \right]-k_{d1}\left[ L_{1}A \right] \right)$ |  |
| $\frac{d\left[ L_{2} \right]}{dt}=-(k_{a2}\left[ L_{2} \right]\left[ A \right]-k_{d2}\left[ L_{2}A \right])$ | (2b) |
| $\frac{d\left[ L_{2}A \right]}{dt}=\left( k_{a2}\left[ L_{2} \right]\left[ A \right]-k_{d2}\left[ L_{2}A \right] \right)$ |  |

The model represents that a FliC-NikR_C_ molecule (ligand) has two kinds of binding sites (*L_1_* and *L_2_*), where each individual site can bind one Ni(II) ion indicated by *A* (analyte). As a result of Ni(II) binding, two types of FliC-NikR–Ni(II) complexes, *L_1_A* and *L_2_A* can be formed in reversible pathways (Eq (1a) and (1b), respectively). *k_a1_* and *k_d1_* as well as *k_a2_* and *k_d2_* correspond to the association and dissociation rate constants when the Ni(II) binds to site *L_1_* as well as *L_2_*, respectively. In the rate equations, the brackets symbolize the molar concentration of the given components. The dissociation constant *K_d_* is determined as the ratio *k_d_*/*k_a_*.

***Note 3: DLS measurement results***

Wild-type flagellin and FliC-NikR_C_ fusion protein have similar sizes containing 494 and 490 amino acids, respectively. Comparison of the particle size distribution of flagellin and FliC‑NikR_C_ by DLS revealed that the latter is not monomeric, it contains small oligomers (**Fig. S1**). The size distribution peak of FliC is centered around 7 nm, while it is shifted to 21 nm in the case of the fusion construct. While the precise state of oligomerization is still uncertain from this experiment, we suppose that FliC‑NikR_C_ may form tetrameric structures similarly to NikR_C_. Attempts to polymerize purified FliC-NikR_C_ failed, presumably due to the oligomerization of subunits interacting with each other through their NikR_C_ domain. According to these observations, the FliC part sterically does not interfere with spontaneous NikR_C_ oligomerization in the FliC‑NikR_C_ fusion protein.


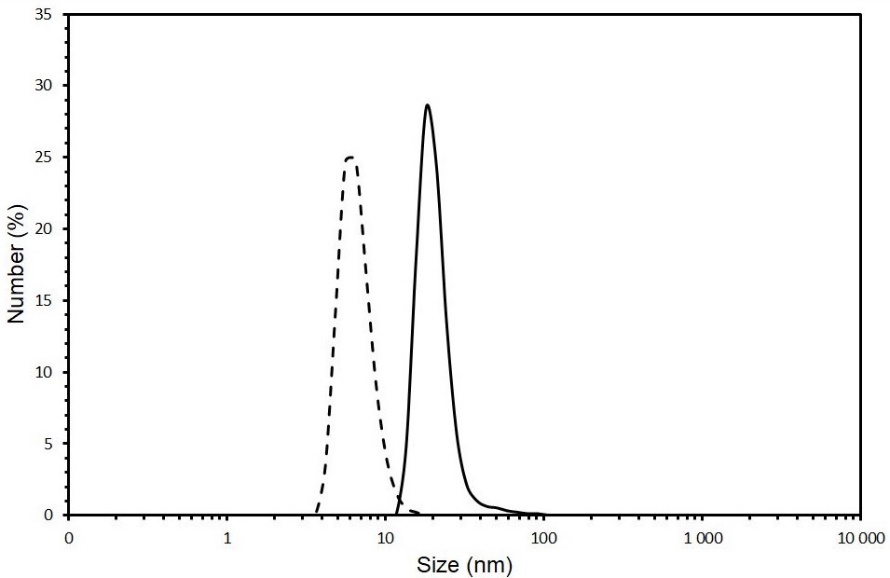


**Figure S1.** Comparison of particle size distribution of flagellin (dashed line) and FliC-NikR_C_ (full line) samples by DLS.

***Note 4: Covalent immobilization on PCH chips: results***

Besides the physisorption of flagellin onto the PCP-LIP surfaces, we also applied covalent immobilization to the hydrogel-based PCH chip that carries a high-capacity 3D immobilization matrix with reactive carboxyl groups. Before the EDC/NHS-based covalent coupling, pH scouting was applied to find the optimal immobilization pH where the repulsion between the negative carboxylate surface and the protein is minimized. **Fig. S2 A** shows a side-by-side plot of FliC-NikR_C_ and FliC injections at different pHs (prepared in acetate buffers). Based on the highest adsorbed mass and similar adsorption level for both proteins, pH 3.5 was selected for covalent immobilization. The observed pH-dependence of the adsorbed masses is related to the isoelectric point of FliC-NikR_C_ and FliC which are 4.79 and 5.25, respectively. The covalent immobilization onto the PCH chip resulted in 4420 and 4700 pg/mm^2^ surface masses of FliC‑NikR_C_ and FliC (**Fig. S2 B-C**).


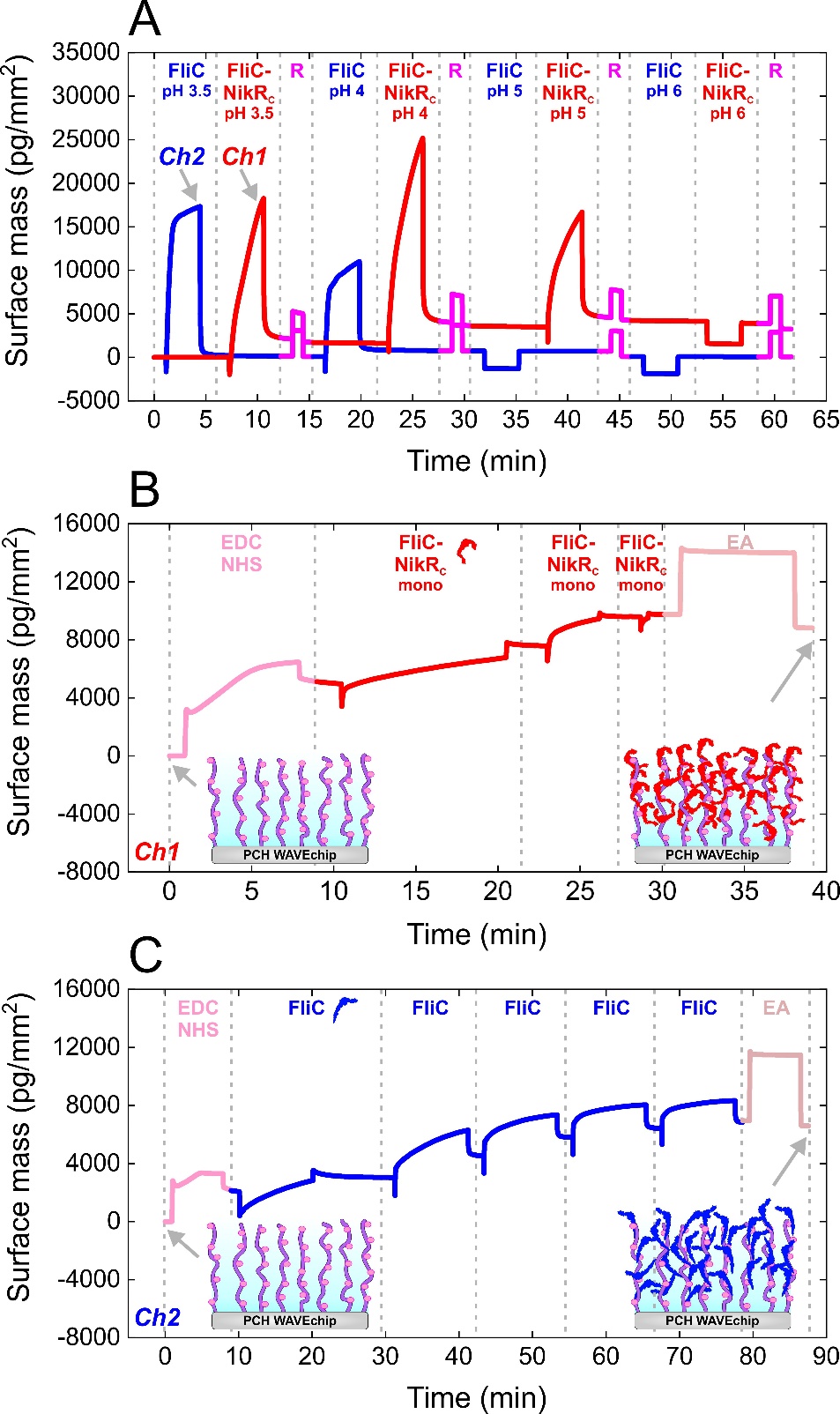


**Figure S2.** **A.** Result of sequential adsorption measurements performed for testing the effect of pH (pH scouting) on the adsorbed amount of FliC-NikR_C_ and FliC flagellins on a PCH WAVEchip. The graph shows the sequential injection of FliC-NikR_C_ (red, *Ch1*) and FliC (blue, *Ch2*) solutions prepared at different pHs. All flagellin injection cycles were followed by a regeneration cycle (R, purple curve) involving the injection of 0.1 M borate, 1 M NaCl, pH 9. **B-C.** FliC-NikR_C_ and FliC immobilization series performed on PCH chip using channel *Ch1* and *Ch2*, respectively. The series started with EDC/NHS injection which was followed by the injection of FliC-NikR_C_ and FliC solution reaching 4420 pg/mm^2^ and 4750 pg/mm^2^, respectively. The series ended with an injection of EA solution. All cycles involve a baseline (RB injection), a flagellin injection and a washing section (RB injection) (cycle sections are not indicated on the graph, but can be followed according to the upgoing/dropping curves as shown in **Fig. 3** in the main text). The schemes on the bottom of the graphs illustrate the thick PCH hydrogel coating and immobilized flagellin molecules. As the applied pH resulted in the decomposition of FliC-NikR_C_ tetramer, monomeric forms are shown.

***Note 5: Summary of GCI results***

**Table S1.** Binding kinetic of Ni(II) to FliC-NikR_C_ protein determined by GCI measurements and kinetic analysis.

|  | High coverage | High coverage | Low coverage |
| --- | --- | --- | --- |
|  | PCP-LIP chip | PCH chip | PCP-LIP chip |
| FliC-NikR_C_ surface mass | 4950 pg/mm^2^ | 4420 pg/mm^2^ | 840 pg/mm^2^ |
| *R*_max1_ | 2.5 pg/mm^2^ | 4.63 pg/mm^2^ | 0.4 pg/mm^2^ |
| *R*_max2_ | 28.4 pg/mm^2^ | 39.31 pg/mm^2^ | 5.1 pg/mm^2^ |
| *k*_a1_ | 5.22 × 10^4^ M^-1^ s^-1^ | 5.92 × 10^3^ M^-1^ s^-1^ | 1.47 × 10^8^ M^-1^ s^-1^ |
| *k*_d1_ | 3.08 × 10^-3^ s^-1^ | 5.02 × 10^-3^ s^-1^ | 8.71 × 10^‑1^ s^-1^ |
| *k*_a2_ | 6.07 × 10^3^ M^-1^ s^-1^ | 3.15 × 10^4^ M^-1^ s^-1^ | 1.51 × 10^3^ M^-1^ s^-1^ |
| *k*_d2_ | 1.43 × 10^-1^ s^-1^ | 1.61 × 10^-1^ s^-1^ | 7.12 × 10^-2^ s^-1^ |
| *K*_d1_ | 59 nM | 0.85 μM | 5.9 nM |
| *K*_d2_ | 23.5 μM | 5.1 μM | 47 μM |
